# Apparent Life-Threatening Events (ALTE): Italian guidelines

**DOI:** 10.1186/s13052-017-0429-x

**Published:** 2017-12-12

**Authors:** Raffaele Piumelli, Riccardo Davanzo, Niccolò Nassi, Silvia Salvatore, Cinzia Arzilli, Marta Peruzzi, Massimo Agosti, Antonella Palmieri, Maria Giovanna Paglietti, Luana Nosetti, Raffaele Pomo, Francesco De Luca, Alessandro Rimini, Salvatore De Masi, Simona Costabel, Valeria Cavarretta, Anna Cremante, Fabio Cardinale, Renato Cutrera

**Affiliations:** 1 Sleep Breathing Disorders and SIDS Center, Meyer Children’s Hospital, Firenze, Italy; 2 Department of Perinatal Medicine, Institute for Maternal and Child Health-IRCCS Burlo Garofolo, Trieste, Italy; 3 Paediatric Department, University of Insubria, Varese, Italy; 4 Department of Neuroscience, Psychology, Drug Research and Child Health, University of Florence, Firenze, Italy; 5 Neonatal Intensive Care Unit, Del Ponte Hospital, Varese, Italy; 6 SIDS Center, Pediatric Emergency Department, “G. Gaslini” Children’s Hospital, Genova, Italy; 7 Pneumology Unit - University Hospital Pediatric Department, Bambino Gesù Children Hospital, IRCCS, Rome, Italy; 8 SIDS/ALTE Center, Buccheri la Ferla Hospital, Palermo, Italy; 9 Pediatric Cardiology, Santo Bambino Hospital, Catania, Italy; 10 Cardiology Unit, G. Gaslini Children’s Hospital, Genova, Italy; 11 Clinical Trial Office, Meyer Children’s Hospital, Firenze, Italy; 12 Emergency Department of Paediatrics, G. Gaslini Children’s Hospital, Genova, Italy; 13 National Neurological Institute IRCCS C, Mondino, Pavia, Italy; 14 Pediatric Unit, Giovanni XXIII Hospital, Bari, Italy

**Keywords:** Apparent life-threatening events, Brief resolved unexplained events, Sudden unexpected early neonatal death, Sudden unexpected postnatal collapse

## Abstract

Five years after the first edition, we have revised and updated the guidelines, re-examining the queries and relative recommendations, expanding the issues addressed with the introduction of a new entity, recently proposed by the American Academy of Pediatrics: BRUE, an acronym for Brief Resolved Unexplained Events. In this manuscript we will use the term BRUE only to refer to mild, idiopathic cases rather than simply replace the acronym ALTE per se.

In our guidelines the acronym ALTE is used for severe cases that are unexplainable after the first and second level examinations.

Although the term ALTE can be used to describe the common symptoms at the onset, whenever the aetiology is ascertained, the final diagnosis may be better specified as seizures, gastroesophageal reflux, infection, arrhythmia, etc. Lastly, we have addressed the emerging problem of the so-called Sudden Unexpected Postnatal Collapse (SUPC), that might be considered as a severe ALTE occurring in the first week of life.

## Background

This document concerning the update of the Guidelines for ALTE has been drawn up 5 years after the 1st edition, on the basis of the same methodological criteria from the National Guidelines Plan- PNLG (Table 1), already adopted for the original document [1].

**Table 1:** Definition of the levels of evidence and the grade of recommendations [1]

EVIDENCE LEVEL	
I = Evidence obtained from several controlled, randomised clinical trials and/or systematic revisions of randomised trials	
II = Evidence obtained from a single randomised trial with an appropriate design	
III = Evidence obtained from cohort studies with controls or their meta-analysis	
IV = Evidence obtained from case-control retrospective studies or meta-analysis	
V = Evidence obtained from case studies (series of cases) without a control group	
VI = Evidence based on the opinion of qualified experts or a committee of experts as indicated in the guidelines, in consensus conferences, or based on the opinions of the members of the work group responsible for these guidelines	
GRADES OF RECOMMENDATION	
A = the performance of that particular procedure or diagnostic test is highly recommended (indicated by a special recommendation supported by good quality scientific evidence, even though not necessarily of Types I or II)	
B = there are some doubts about the fact that this particular procedure/operation must always be recommended, however it is considered that its performance must be physical examined	
C = there is substantial uncertainty in favour of or against the recommendation to perform this procedure or operation	
D = the performance of this procedure is not recommended	
E = the performance of this procedure is strongly advised against	

A Working Group assessed the adequacy of the answers to the clinical queries already included in the previous version of the document and added some ex-novo queries.

A new chapter has been added relating to early neonatal ALTE, also known as Sudden Unexpected Postnatal Collapse (SUPC).

Moreover, according to the recent suggestions from the American Academy of Pediatrics, a new acronym has been added: BRUE, i.e. Brief Resolved Unexplained Events, which refers to mild idiopathic cases.

Each clinical query has been answered on the basis of bibliographical research.

The research filters were applied to the PubMed Database.

The papers retrieved were screened in order to verify their pertinence to the clinical query.

Each paper included has been subjected to a quality evaluation and the most relevant results used for the purpose of drawing up the recommendations.

The summary of the literature search over the last 5 years, its consistency with what was already drafted in the previous version of the document, and the final draft of the recommendations were discussed with the members of the panel.

This document is therefore the result of a process of sharing and contextualisation of the updated scientific literature on ALTE.

The need for the development of new recommendations on ALTE emerged when the database on ALTE cases from the Italian Paediatrics Society (SIP) website was made available [2]. Consequently, an ad hoc Working Group on ALTE was set-up and organized into sub-committees.

## Clinical queries

1. Is the acronym ALTE still valid?
2. What is the real incidence of these episodes?
3. What can cause an ALTE?
4. What features of the medical history should be collected?
5. How is a correct physical examination performed?
6. What first-level examinations should be performed?
7. What second-level examinations should be performed?
8. When should an infant with a history of ALTE be hospitalised?
9. Is hospital cardiorespiratory monitoring always indicated for infants with ALTE?
10. Which are the indications for home monitoring of infants with ALTE?
11. What are the complications of ALTE?
12. Which need for a follow-up after an ALTE episode?
13. Is there a connection between ALTE and Sudden Infant Death Syndrome (SIDS)?
14. What does Sudden Unexpected Postnatal Collapse (SUPC) mean?
15. What are the risk factors for SUPC?
16. How the risk of SUPC can be reduced?

### Implementation strategy

The Guidelines (GL) will be accessible on the website of the SIP.

Moreover, dissemination of the GL will be facilitated after publication in an international peer-reviewed journal.

Ad hoc training courses are expected to complete the dissemination process.

### Monitoring and assessment

The health burden due to cases of ALTE will be assessed on the basis of the following indicators:

- number of hospital admissions due to ALTE per total number of admitted infants, and length of stay
- number of re-admissions
- number and type of lab tests and instrumental workup
- number of infants prescribed home monitoring at hospital discharge
- outcome at 1 year follow-up

Adherence of paediatricians to current GL in the management of ALTE will be assessed before and after dissemination of the GL on a selected sample of centres dedicated to the management of ALTE.

## Definition

The acronym ALTE is understood, in an infant under the age of 1 year, as: “*an episode that is frightening to the observer and is characterised by some combination of: apnoea (central or occasionally obstructive), colour change (usually cyanotic or pallid, but occasionally erythematous or plethoric), marked change in muscle tone (usually marked limpness), choking or gagging. In some cases the observer fears that the infant has died”.*

Previously used terminology such as “aborted crib death” or “near-miss SIDS” has been abandoned as possibly misleading to a close association between ALTE and SIDS [3].

## Query 1

### Is the acronym ALTE still valid?

The acronym ALTE (Apparent Life-Threatening Event) is widely used in medical literature, sometimes particularly referring to acute and severe events (Acute Life-Threatening Episodes or Events) [4, 5], Nevertheless, the American Academy of Pediatrics (AAP) has recently proposed the replacing of the term ALTE with the new acronym BRUE (Brief Resolved Unexplained Events) [6] to allow for more easily classifying and managing all those patients who come under medical observation without any symptomatology and with a clinical history that suggests minor episodes.

This new classification would be intended to single out from the heterogenous group of ALTE of non-severe, idiopathic episodes lasting ≤1 min, which require just an accurate history and correct clinical assessment. The recent GL of the AAP practically exclude the higher risk episodes. Specifically, BRUE are characterised by one or more of the following symptoms:

1. cyanosis or pallor (erythrosis has now been excluded)
2. absent, decreased or irregular breathing;
3. marked alteration of muscle tone (hypo/hypertonia);
4. altered level of responsiveness.

The present GL refer to more severe episodes only solved after stimulation manoeuvres, for which the old acronym ALTE, is more appropriate.

### Recommendation 1

The acronym ALTE should be maintained for the severe idiopathic episodes, while acquiring the new acronym BRUE for the lower-risk idiopathic episodes.

**Evidence level VI**

**Grade of recommendation A**

## Query 2

### What is the real incidence of ALTE?

The precise incidence of ALTE is not known from the available scientific literature. This depends mainly on the different clinical interpretation given to these episodes, on the substantial inequality in the clinical/instrumental approach, on the variable coding of the event, and on the fact that the studies refer mainly to cases arriving under hospital observation.

In some retrospective studies [7, 8] a very high incidence is reported (4-6%) but it could be falsified by *recall bias*; in addition, these data are based exclusively on questionnaires administered to the parents, without documented medical observation of the infants. Nevertheless, even by excluding these studies, the real incidence of ALTE is hardly definable as the figures reported swing between approximately 0.5 and 10‰ [9, 10].

More recent prospective studies [11, 12] indicate an incidence ranging between 0.58 and 2.46/1000 live births.

Certainly, ALTE represent quite a common event in the emergency departments, reaching 0.6-0.8% of the total admissions of infants [13].

As soon as two different codes are available for ALTE and BRUE, the distinction between the incidence of higher-risk cases and lower-risk ones will be possible and recorded for epidemiological purpose.

## Query 3

### What can cause an ALTE?

Clinical workup only allows for identifying a specific aetiology in 50-70% of cases [14], in which the final diagnosis will no longer be ALTE, but rather it will pinpoint the recognized cause that has triggered the event, i.e. gastroesophageal reflux, seizures, infections, etc... The remaining cases are represented by the so-called idiopathic ALTE (IALTE), which represent approximately 15% of the total cases.

In order to stabilise the infant, the witnesses report having to carry out vigorous resuscitation manoeuvres, at times even resorting to cardiopulmonary resuscitation in the case of intervention by healthcare professionals.

The most frequent diseases causing episodes having the features of ALTE are represented by digestive tract diseases, [14–16] neurological diseases, respiratory tract infections, endocrine-metabolic diseases, cardiac diseases and child abuse [12, 14, 15, 17].

Two reviews have recently been published [18, 19], as well as two studies, one of which conducted on infants with ALTE admitted to the emergency department [20], and the other, a survey carried out in the Netherlands in second and third level hospitals [12].

Summarizing, these papers highlight that:

- More than 80% of the infants admitted to the emergency department do not present any severe conditions and a specific diagnosis is only obtained in about 30% of cases [20].
- Approximately 50-58% of the cases classified as ALTE can be associated with co-morbidities: gastroesophageal reflux, seizures, and infections of the lower airways. Other associated causes include: breath-holding spells, arrhythmias, congenital heart diseases, meningitis, ingestion of drugs like codeine, or poisoning [12, 18, 19].

## Gastroesophageal reflux

To start with, two main issues are worth clarifying: a) how relevant is the gastroesophageal reflux (GER) among the causes of ALTE, and b) how accurate is the diagnosis of GER?

### The relationship between GER and ALTE

Diseases of the digestive tract and GER have long been considered a major cause of ALTE [21–24] but its relation is still controversial.

Wenzl et al. [25], using polysomnographic recordings simulatenously with esophageal pH-impedance, first demonstrated a close correlation between both acid and non-acid GER and episodes of apnoea.

More recently, an assessment of 58 preterm infants investigated for recurrent apnoea with pH-impedance monitoring and simultaneous polysomnography (PSG), reported a greater frequency of apnoea after acid and non acid GER episodes than during the periods preceding GER or those without reflux [26]. A Spanish study of 39 infants with ALTE showed underlying GER in 33 cases with the combined use of esophageal pH-impedance, and in only 14 with the sole pH-metry analysis, underscoring a greater frequency of non acid GER episodes and the benefit of a combined investigation [27]. Another study in 20 preterm infants (10 with ALTE and 10 controls) with simultaneous pharyngoesophageal manometry, respiratory plethysmography and the use of nasal thermistors suggested a possible role of esophageal motility. The analysis showed more frequent and prolonged spontaneous respiratory events (SRE, defined as apnoea >2″ with >/= 2 “missing” breaths) in patients with ALTE, as well as minor amplitude of the protective contraction reflexes of the upper esophageal sphincter, a greater frequency of altered esophageal propagation, and more frequent mixed apnoea and gasping [28].

On the contrary the European and North American Guidelines on the Clinical Management of GER [29] have questioned the relationship between apnoea and/or ALTE and GER. In fact, the respiratory manifestations of ALTE have been attributed to an exaggerated laryngeal chemoreflex aimed at preventing inhalation of the gastric contents in the respiratory tract, rather than to a true apnoea.

A systematic revision [30] further highlighted the current limited data in literature on the association between GER and apnoea mainly because of the low number of patients recruited, the different inclusion criteria, diagnostic methods and treatment outcomes. Even less studies properly assessed the relationship between ALTE and GER.

### How accurate is the diagnosis of GER?

First of all, regurgitation as an isolated sign of GER should be considered a physiological, functional, and transient phenomenon in infants with spontaneous recovery after the first month of life. Consequently, it is a common manifestation in healthy infants and does not require any investigation or pharmacological treatment, but simply parental education (with feeding modifications if needed) and reassurance.

Similarly, it is evident that the concurrent occurrence of any respiratory symptoms does not imply per se a suspicion of GER-disease (GERD), although some airway diseases (i.e. laryngomalacia, laryngitis, asthma) may lead to secondary GER due to negative intrathoracic pressure or increased abdominal pressure caused by cough.

Hence, if an episode of ALTE is associated with regurgitation, it does not require treatment or investigation for GERD unless severe or recurrent episodes occur, as pointed out in GER GL [29] and in a recent systematic review on the approach to ALTE [31]. As no specific symptom exists and regurgitation is neither specific nor necessary for the diagnosis of GERD, in the selected cases, it is recommended to perform 24-h pH-impedance with simultaneous PSG recordings to quantify acid and non acid GER and to detect possible relation with apnoea [29, 31, 32]. Ultrasound, X-Ray and scintigraphy have low sensitivity and/or specificity for GER and do not allow a symptom association assessment [29].

Methodology, indications and interpretation of esophageal pH-impedance monitoring [33], limitations [32, 34] and pediatric reference values [35, 36] have been recently published. Indications for upper endoscopy are listed in Table 2.

**Table 2:** Recommended diagnostic procedures for GER/GERD focusing only on infants with apnoea or ALTE [37]

Diagnostic investigation	When to perform it
pH-monitoring, or better, pH- impedance if available	- suspected recurrent aspiration pneumonia - unexplainable apnoeas or inflammation of the upper airways or non-epileptic simil-convulsive episodes or apparent Sandifer Syndrome
Upper endoscopy	- reported haematemesis not caused by ingested blood - persistent regurgitation after the first year of life - prolonged refuse to eat or persistent poor growth associated with regurgitation - unexplainable iron-deficiency anaemia - suspected Sandifer Syndrome

After reviewing 13 papers, the recent National Istitute for Health and Care Excellence (NICE) guidelines on GER [37] confirm how GER is only very rarely the cause of apnoea or ALTE, thus recommending to perform a gastroenterological assessment only in selected cases (Table 2).

No evidence supports the use of acid-inhibitors as diagnostic test or empirical treatment in infants with apnoea or ALTE, whereas these drugs may instead increase the risk of infections [37].

Despite investigation for GERD should be reserved to very few selected infants, if frequent, persistent and troublesome regurgitations emerge from the history of infants, it is advised to provide parents education on correct position and feeding to avoid prone position during sleep, overfeeding, exacerbations of GER and possible recurrent respiratory associated episodes [29, 37]. Alginate treatment for 2-4 weeks can also be considered in troublesome regurgitating infants before performing investigations for GER [37].

In view of the above data, we therefore propose the following recommendations:

### Recommendation 2

Esophageal pH-monitoring for 24 h is recommended for measuring the degree of acid GER present in the esophagus.

**Evidence level I**

**Grade of recommendation A**

### Recommendation 3

pH-impedance monitoring represents the gold standard for detecting any type of GER (acid or non-acid) and for assessing the temporal association between symptoms and reflux. pH-impedance monitoring is recommended in infants with persisting troublesome GER symptoms and/or recurrent respiratory manifestations.

**Evidence level I**

**Grade of recommendation B**

### Recommendation 4

While the oesophagus-gastric ultrasound is a very sensitive investigation (95%), it is scarcely specific (11%) and has too short a duration to make an accurate assessment of the association between GER and symptoms.

Therefore it cannot be recommended in the diagnosis of GER.

**Evidence level I**

**Grade of recommendation D**

## Neurological diseases

According to various authors, diseases of the nervous system account for 9-30% of ALTE [14, 15, 38].

Seizures, both isolated or caused by intracranial bleeding, hydrocephalus or hypoxic damage, are recognised as the most common neurological symptoms in cases of ALTE.

In a retrospective study it is reported that epilepsy is the cause of ALTE to a greater extent than febrile seizures or other non specific and isolated episodes; moreover, in 3.6% of ALTE, seizures represent the symptom of the onset of epilepsy [39].

The time elapsing between the first episode of seizures causing the ALTE and a possible recurrence is short, being 1 week in about 50% of cases and 1 month in more than 70% of cases [40], calling for cautious administration of an anti-epileptic therapy and close follow-up of infants.

Conversely, we must consider that seizures may not be the cause of ALTE, but, instead could be secondary to hypoxaemic episodes that occur during ALTE [41, 42].

Other neurological causes of ALTE include infections of the Central Nervous System (CNS), intracranial hypertension (secondary to tumours, subdural haematomas, and metabolic diseases), malformations of the CNS, and neuromuscular diseases.

The clinical history and examination are of pivotal importance for raising the suspect of a neurological disease associated to ALTE. When required, the entire clinical-instrumental neurological assessment may include an electroencephalogram (EEG), video EEG, cranial ultrasound, fundoscopic examination, cerebrospinal fluid, evoked potentials, and Magnetic Resonance Imaging (MRI) [19, 43, 44].

### Recommendation 5

The performing of an EEG is strongly recommended in case of a suspected neurological disease or in the event of recurrent ALTE.

**Evidence level III**

**Grade of recommendation A**

### Recommendation 6

Neurological consultation and the performing of an EEG are not recommended in an infant at the first episode of ALTE, in the absence of a clinical history and/or neurological signs.

**Evidence level IV**

**Grade of recommendation D**

## Infectious diseases

Infectious diseases, and among these, respiratory tract infections, are quite a common cause of ALTE (8-15%) [14, 45, 46].

Pertussis and urinary tract infections are more frequently involved than meningitis or sepsis. ALTE prevail in infants under the age of 60 days, in whom scarce clinical symptomatology and the absence of fever may delay the therapeutic approach, thus increasing the risk of a rapid deterioration of the vital functions [47].

The microorganisms most frequently involved are Respiratory Syncytial Virus (RSV), Mycoplasma pneumoniae, *Haemophilus* influenzae, *Bordetella pertussis* and the Influenza and Parainfluenza viruses. RSV is one of the main causes of apnoea in infants [48]. Similarly, *Bordetella pertussis* may be the cause of ALTE in infants who have not yet been immunized [49].

Urinary tract infections (UTIs) represent approximately 1% of the infectious diseases that cause ALTE.

Initial lab tests in case of an ALTE due to a suspected infectious disease include a whole blood count (WBC), C-reactive protein (CRP) and urine analysis and culture, according to Italian and AAP GL on UTIs [50].

In case of suspected respiratory tract infection, it is recommended to carry out a rapid test for RSV [15, 45, 46] and when low respiratory tract infection is suspected the performing of a chest x ray is recommended [51–55].

### Recommendation 7

The performing of a series of basic laboratory examinations (WBC, CRP) and a urine analysis and culture is recommended in cases of ALTE from a suspected infectious disease.

**Evidence level V**

**Grade of recommendation B**

### Recommendation 8

In case of respiratory tract infections, it is recommended to carry out tests for RSV and *Bordetella pertussis*.

**Evidence level III**

**Grade of recommendation B**

### Recommendation 9

In case of a suspected infection of the lower airways, it is recommended to carry out a chest X-ray.

**Evidence level I**

**Grade of recommendation A**

## Non-infectious respiratory diseases

### Obstructive sleep Apnoea syndrome

Between 4 and 10% of ALTE are ascribable to respiratory obstructions during sleep that may in time turn into real Obstructive Sleep Apnoea Syndrome (OSAS) [14].

Some infants who die from SIDS show a higher number of obstructive apnoea during sleep [56] and in some family strains with a history of OSAS, a higher incidence of both ALTE and SIDS has been reported [57–60]. Consequently, PSG and/or ear, nose and throat (ENT) consultation (including a fiberoptic rhinolaryngoscopy) might be indicated in selected cases [14].

During the first months of life, apnoea may be obstructive, usually associated with respiratory infections, or alternatively, it may be idiopathic, especially in former preterm infants. As many as 58% of cases in a cohort of 348 infants aged between 3 weeks and 3 months were attributable to an obstructive apnoea (e.g. due to mandibular retrognathia). The presence of thoracic/abdominal asynchrony (paradoxical breathing) during sleep has also been described, which is an expression of the increase in the respiratory effort needed to overcome the increased resistance at the level of the upper airways [61].

A recent study [62] conducted on preschool children with a previous history of ALTE has demonstrated a greater frequency of respiratory disturbance in these children during sleep than in controls, emphasising the importance of the PSG also in these cases.

In infants with ALTE, the PSG is only indicated when there is the clinical suspicion of a respiratory sleep disorder [63].

### Recommendation 10

The PSG is recommended in all cases of ALTE when a respiratory sleep disorder is suspected.

**Evidence level I**

**Grade of recommendation A**

### Breath holding spells

Around 7% of ALTE are attributable to Breath Holding Spells (BHS) [64] which are classically divided into cyanotic, pale and mixed forms. The cyanotic forms of BHS are characterized by a prolonged expiratory apnoea followed by a rapid central cyanosis due to severe hypoxaemia. The cause of cyanotic BHS has been attributed to a right-left intrapulmonary shunt which would explain the rapid onset of hypoxaemia, rather than the apnoea itself [65].

BHS may be triggered by prolonged crying or by a sudden and/or painful stimulus; approximately 25% of these episodes occur before the age of 6 months and 66% before the age of 1 year [66]. The diagnosis of BHS in the context of the ALTE episodes does not imply particular difficulties. In case of BHS, the clinician should be aware of the association between iron-deficiency anaemia and BHS which makes it advisable to perform a FBC and analysis of the serum iron level [67–69].

Generally, BHS clear up spontaneously after the first 2 years of life, and based on the data in literature, neurological sequelae can be ruled out [65].

### Recommendation 11

Performing a WBC and an assessment of the serum iron levels are recommended in case of BHS.

**Evidence level IV**

**Grade of recommendation B**

### Congenital central hypoventilation syndrome

In extremely rare cases, ALTE may represent the onset of a Congenital Central Hypoventilation Syndrome (CCHS).

The CCHS, also known as Ondine’s Curse, is a rare and complex disease characterised by impairment of the Autonomic Nervous System (ANS) and in particular, the automatic control mechanisms of breathing during sleep. The prevalence is about 1 case every 200,000 births. It is a genetically-based disease and in 90% of cases the defect is represented by the heterozygote mutation of the *PHOX-2B* gene [70].

The genetic alteration determines a complex disease dominated by the impairment of the ANS in which there is a severe respiratory depression during sleep, so-called *forgotten breathing*, indicating the failure of the respiratory drive during sleep. Generally, the diagnosis is made in the first days/weeks after birth because these infants show a severe respiratory insufficiency not attributable to common neonatal diseases.

However, there may also be a later-onset form characterised by a more subtle symptomatology, that may manifest as ALTE.

The diagnosis of CCHS is suggested by documented severe and progressive hypercapnia and hypoxaemia that take place during sleep, in the absence of pulmonary and neuromuscular diseases. The PSG demonstrates the hypoventilation and the diagnosis is confirmed by genetic test.

### Metabolic diseases

The episodes of ALTE due to congenital errors of the metabolism vary between 2 and 5% [14]. The ß-oxidation defects of fatty acids (Acyl-CoA dehydrogenase deficiency), defects of the urea cycle, galactosaemia, Reye Syndrome, and nesidioblastosis are common metabolic diseases involved in ALTE.

A metabolic disease must be suspected in infants with a clinical history of ALTE in case of recurrent idiopathic events and/or sudden unexpected deaths in the family. An ALTE may represent either the onset or the decompensation of a metabolic disease. The most frequent symptoms are represented by: apnoea, hypertonia, hypotonia, seizures, vomiting and failure to thrive.

In these cases, it is recommended to assess the acid-base balance, to perform blood glucose and ammonia analyses and a full metabolic screening. A specialist assessment is recommended when an in-depth diagnostic analysis is required [71].

### Recommendation 12

When a metabolic disease is suspected, acid-base balance, blood glucose, ammonia, serum lactic acid, urine organic acids, plasma amino acids and plasma acylcarnitine analyses are recommanded. A specialist assessment is indicated in the event of symptoms and/or first tests requiring an in-depth diagnostic procedure.

**Evidence level I**

**Grade of recommendation A**

## Heart diseases

These may represent 0.8-3% [15] of the causes of ALTE [14].

Anomalies in the heart rhythm such as Wolff-Parkinson-White Syndrome and the long-QT Syndrome are the most frequent heart anomalies. Congenital cardiac malformations, myocarditis and cardiomyopathies instead represent a less frequent cause.

The prolongation of the Q-T interval of the electrocardiogram (ECG) is related to a higher risk of SIDS [72]. Particular conditions such as strong emotion or sleep, may expose the infant to potentially fatal arrhythmias and/or syncopal or pre-syncopal episodes [73, 74]. The prevalence of the long Q-T syndrome, as revealed by an Italian multicentre study, is about 1/2500 live births [75]. In one study [45] it was found that an ECG should not be recommended in all infants with a clinical history of ALTE, since the diagnostic yield of this examination seems to be extremely low. In a wide retrospective study of the clinical charts of 2179 patients with a history of ALTE conducted in 43 American hospitals [76], the ECG was carried out in approximately 50% of the cases (range 0%-93%), highlighting a pronounced lack of homogeneity in the diagnostic approach to these patients.

In a retrospective study conducted on 485 patients with a history of ALTE [77], the prevalence of heart diseases was around 4%, of which 1% was relevant. As ECG has a high percentage of false positives, this could have a negative impact on the family and ultimately represent a financial burden for health care systems. In view of the risk of failing to identify potentially lethal diseases, often of genetic origin, this test is recommended in all cases of ALTE. In accordance with the recommendation formulated by the AAP, this examination may at times be recommended in cases of BRUE as well.

The Holter ECG is not considered a first-level examination, however, it must necessarily be conducted in cases of a brady- or a tachycardia detected by the ECG and/or at physical examination.

### Recommendation 13

The performing of an ECG is recommended in all infants with a history of ALTE.

**Evidence level VI**

**Grade of recommendation A**

## Idiopathic ALTE

IALTE were classically considered synonymous with Infantile Apnoea, that is, episodes of apnoea that occur in newborns after the 37th week of post-conceptional age (PCA), the time of the theoretical completion of maturation of the bulbar respiratory centres. This is misleading as a great deal of evidence demonstrates that the nature of ALTE is very complex and still substantially unclear: a respiratory control disorder is most likely accounting for the particular “instability” of these infants [3].

The main alterations involved in the pathophysiology of IALTE can be summarized as follows:

1. Alterations of the respiratory drive, such as reduced responsiveness to hypoxic and hypercapnic challenges [78], a greater number of apnoeic pauses [79], an increase in periodic breathing [80], and malfunctioning of the brainstem nuclei involved in respiratory control during sleep [81]. Some peptides contained in cow’s milk have an opioid-like activity. Low levels of DPPIV (dipeptidyl peptidase IV), which is the enzyme involved in the metabolism of these peptides has been found in the serum of infants with ALTE, and it could be at the basis of an opioid-induced respiratory depression [82].

2. Alterations of the respiratory function such as reduced airway conductance and/or maximum flow at functional residual capacity (Vmax FRC) [83], the increase in the phase angle and a greater number of hypoxaemic episodes during sleep [84]. The mismatch in the ventilation/perfusion detected by means of cardiorespiratory recordings (including the detection of transcutaneous oxygen tension (P_tc,O2_), thoracic movements, ECG, heart rate and oxygen saturation S_p,O2_), during cyanotic spells in infants with ALTE have made it possible to shed light on the rapid onset of hypoxaemia, the presence of abrupt hypoxaemic episodes even in the presence of ventilation, the different rapidity of the onset of hypoxaemia in the different types of apnoea and the simultaneous onset of apnoea and hypoxaemia. These phenomena would be explainable in pathophysiological terms by the existence of a right-left pulmonary shunt in patients with reduced airway patency [61].

3. Alterations in the sleep architecture [85]. The increase in the arousal threshold and a greater fragmentation of non-REM sleep compared to controls [86].

4. Alterations in the upper airways in craniofacial syndromes capable of compromising breathing during sleep [59, 62], obstructions at the laryngeal level in preterm newborns [87], reduced development of the mandible [61, 88] and a high incidence of OSAS in the family with multiple cases of SIDS/ALTE [60].

5. Alterations of the ANS demonstrated by a reduction in the cardiac response to the tilt-test and postural hypotension, alterations in the regulation of blood pressure, the increase in the arousal threshold in infants with ALTE attributable to OSAS [89], and vagal hypertonia [64].

6. Genetic alterations. These represent an intriguing aspect since common alterations have been found in the gene that codifies for the serotonin transporter (5HTT) in infants who have died of SIDS and in infants with ALTE [90].

In short, there are still many questions on the pathophysiology of ALTE which, despite a “stereotyped” manifestation, could be the expression of the multiple diseases we have described above, or else remain unexplainable.

Even if initially classified as ALTE, when a specific disease has been defined, it should be coded accordingly.

In the most severe idiopathic cases it is appropriate to keep to the original definition of ALTE, an acronym that clearly describes the danger of the event that remains unexplainable after adequate workup.

## Query 4

### What features of the medical history should be collected?

The history collection represents the first step in the diagnostic process. In fact, the infant is usually taken to the paediatrician or the emergency department (ED) after overcoming the event and when he/she is in good conditions. The information must be provided above all by the witnesses of the event, those who have provided first aid and subsequently the parents if they were not present at the time of the episode.

After the first data collection, which takes place at a time of stress for the witnesses of the event, there must be an in-depth investigation during the following hours [14, 91].

The family history must specifically refer to any possible presence of ALTE/BRUE or sudden death in the collaterals, as a familiarity is detectable in respiratory, cardiac or metabolic diseases.

The pre-perinatal history must include the gestational age and birthweight since both prematurity and low gestational weight represent risks factors [46].

With the past medical history, any diseases already diagnosed will be considered as they could be associated with the risk of sudden death or a recurrence of the episode.

With the recent history, attention must be paid to the clinical conditions of the infant during the 24 h prior to the episode (presence of fever, vomiting, diarrhoea or feeding difficulties), to any immunizations carried out (possible syncopal reactions), to the introduction of any new foods or medication (possible anaphylactic reactions), to the type and method of breastfeeding, to any drugs taken by the mother if nursing (for example codeine or diazepam), or to changes in the feeding schedules (possible triggering factor of hypoglycaemia in case of latent metabolic diseases). Stress deriving from abrupt changes in the infant’s habits, such as sleep deprivation on occasion of flying, holiday or weekend trip, etc., is also considered a possible triggering factor [14, 92].

Possible drug administration, either accidental or intentional, should always be carefully searched for and possibly confirmed by urine and blood toxicological tests.

The history of the event is a crucial step in the diagnostic procedure. The medical interview with the observer must therefore aim at obtaining a very detailed collection of information on the clinical conditions of the infant in relation to his/her:

- Behavioural state (asleep or awake)
- Skin colouring (pallor, cyanosis, erythrosis)
- Muscle tone (hypo- or hypertonia)
- Breathing (apnoea characterized by the absence of chest movements, signs of distress, gasping, noisy breathing)
- Position (supine, prone, on side or in adult’s arms)
- Associated symptomatology (regurgitation, vomiting, atypical crying)
- Environmental conditions (cigarette smoking, ambient temperature, clothing, bed covers, presence of gas oven or boiler, fireplace or wood stove)
- Sleeping habits (rooming-in, bed-sharing, sleeping surface)

The endpoint of the medical interview refers to the resuscitation intervention carried out, as this may vary from mild tactile stimulation to cardiopulmonary resuscitation performed by the observer and/or the emergency service. The more severe ALTE occur while sleeping, manifesting with extreme pallor, marked hypotonia and apnoea, and may require vigorous resuscitation manoeuvres (intubation, pharmacological resuscitation) to be resolved [93].

Table 3 gives a detailed picture of the history collection procedure.

**Table 3:** Useful anamnestic data in the assessment of a potential ALTE [12, 16, 44, 84]

Family history	
History of SUDI or SIDS	
Cases of ALTE/BRUE in family members	
Heart diseases (arrhythmias, long-QT syndrome, syncope)	
Hereditary/genetic/metabolic diseases	
Allergic diseases	
Epilepsy, breath-holding spells, delayed growth	
Malformations (craniofacial, musculoskeletal)	
Past medical history	
Pre-perinatal history	
Prematurity	
Birth	
Neonatal weight	
APGAR score	
Normal neonatal screenings	
Breastfeeding	
Complementary feeding	
Height-weight increase	
Adequate psycho-motor development	
Heart diseases	
Neurological diseases	
Previous episodes of ALTE/BRUE	
Feeding problems (GER)	
Sleep and respiratory disorders during sleep (snoring, apnoea)	
Respiratory disorders while awake (noisy breathing)	
Trauma, emergencies	
Previous hospitalisation, surgery	
Immunizations	
Drug assumption	
Other	
Recent history	
Infant’s general condition over the last 48 h (respiratory tract infections, immunizations, fever, other)	
Injuries, falls, recent unexplainable bruising	
Drug assumption	
Introduction of new foods	
Change of feeding schedule	
Sleep deprivation	
Alteration of normal sleeping-wake pattern and/or lethargy	
History of the event	
General description	
The person reporting the event	
Witness of the event (parents, other children, other adults)	
Reliability of the narrator	
Circumstances of the event	
Where it happened (at home or elsewhere; in bed, in cot, on sofa, on floor, etc.)	
Position: supine, prone, erect, sitting, in movement	
During sleep: indicate whether infant emitted sounds immediately beforehand, had noisy breathing, coughed, vomited, etc.	
While awake: indicate whether immediately beforehand the child coughed, vomited, cried in an unusual manner, went stiff, or breathed in food	
While feeding	
During bath time	
Time elapsed since last feed	
Environmental risk factors: cigarette smoking, carbon monoxide, ambient temperature, clothes, objects too close to the infant, noises, accidental causes	
Psychological factors	
Infant’s appearance during the event	
Skin colour: pale, cyanotic, erythrosic, ashen, marbled	
Colour of the lips: normal, pale, cyanotic	
Muscle tone: normal, hypotonia, hypertonia	
State of consciousness	
Jerking of the limbs	
Body temperature: hypo-hyperthermia	
Profuse or absent sweating	
Respiratory distress	
Apnoea	
Bleeding	
End of the event and actions taken	
Time elapsing between the onset of symptoms and first intervention	
Time elapsing between intervention and resumption of breathing and regaining of normal appearance and behaviour	
Did the episode clear up spontaneously or after stimulation?	
Did the episode clear up after vigorous and prolonged stimulation or resuscitation manoeuvres?	
Did the episode end abruptly or gradually?	
Were the resuscitation manoeuvres carried out by parents or by others?	
Before returning to normal was the child calm, confused, agitated, irritable, crying?	
Was the emergency service called?	
Socio-Environmental History	
Family structure, independent home (one or more families)	
Home in good condition (no mould, etc.)	
Recent changes, stressful conditions or internal conflict	
Exposure to toxic substances and drugs	
Need to access social services	
Level of family anxiety	
Contact of the infant with adults with a history of mental illness or substance abuse	
Indexes of suspected or possible abuse	
Previous assistance by the social services of the juvenile court (domestic violence, abuse)	
Repeated changes in the version recounted of the circumstances of the event	
History of unexplainable bruises	
History incompatible with the infant’s psychomotor development	
Incongruence between the observations of the person taking care of the infant and the latter’s psychomotor development, with the infant blamed for bad behaviour	

The importance is reiterated of obtaining specific and punctual information about the clinical history and of carrying out a complete and thorough physical examination of the child has been recently reiterated by Sahewalla [18]. Nevertheless, after obtaining the full history, performing a careful physical examination and some basic laboratory investigation, the clinician may be able to identify the causes of ALTE in a limited number of cases [19].

### Recommendation 14

A targeted and accurate medical interview is essential for ensuring a correct classification of the infant with ALTE.

**Evidence level V**

**Grade of recommendation A**

## Query 5

### How is a correct physical examination performed?

Besides the interview, an in-depth objective examination of the infant represents the other fundamental step in the definition of any suspected case of ALTE.

As for the history collection, the physical examination should also be repeated twice, several hours after the emergency access; this is facilitated if the patient is admitted to hospital or kept under observation.

We recommend to accurately assess the patient’s physical conditions (Table 4), recording the vital functions, the neurobehavioral status, any signs or symptoms of infection, injury, abuse or dysmorphisms.

**Table 4:** Objective examination for the assessment of a potential ALTE [43, 46, 47]

• Weight, length, head circumference • Monitoring of the vital functions: heart rate (HR), respiratory rate (RR), blood pressure (BP), skin temperature, S_p,O2_ • Type of crying • Respiratory function	
• Skin colour, skin perfusion (capillary refilling time)	
• State of hydration • Eye assessment (extra-ocular movements, pupillary response, conjunctival bleeding, examination of the retina if indicated) • Examination of ear and oropharynx (nasal congestion and secretions, blood in nostrils or oral cavity, evidence of injuries or obstructions, tearing of frenulum) • Search for possible craniofacial dysmorphisms (mandible, jaw, nose) • Assessment of the neck mobility	
• Peripheral pulses, rhythm, rate, heart auscultation • Physical examination of the abdomen (organomegaly, masses, abdominal distension), inspection of genitals	
• Anterior fontanel tension, shape of head, bruises or other lesions • Palpation of ribs for possible yielding, crepitus, irregularities	
• Limbs: muscle tone, lesions, deformities with possible fractures	
• Neurobehavioural assessment, muscle tone, reflexes • Neurological assessment (reactivity to external stimuli, response to sound and visual stimuli, general tone, pupillary reflexes to light, symmetry of the movement, tone and strength) • Presence of meningeal symptoms	

### Recommendation 15

A thorough physical examination is essential for identifying possible causes of ALTE.

The physical examination of hospitalized patients should be repeated, with particular attention to the neurological assessment.

**Evidence level V**

**Grade of recommendation B**

## Query 6

### What first-level examinations should be performed?

Due to the great variety of causes of ALTE, it is not possible to recommend a set of “basic examinations” to be carried out in all cases [14, 92]. The initial investigations must be aimed first and foremost, as with any infant arriving in the Emergency Service for an acute event, at excluding any life-threatening diseases for the infant or which could have results later on if they cannot be treated immediately.

Based on the current literature, and in view of the very young age of the patients, as well as the huge number of diseases that could present as ALTE (728 in one systematic study) [15] and the parents’ anxiety, most authors suggest a panel of investigations in every single case of ALTE.

The first-approach examinations are mandatory whenever the infant appears unstable and/or in cases that have required resuscitation manoeuvres.

For the clinical management of infants with BRUE, reference must be made to the GL of the AAP [6].

### Recommendation 16

**Fig. 1:**
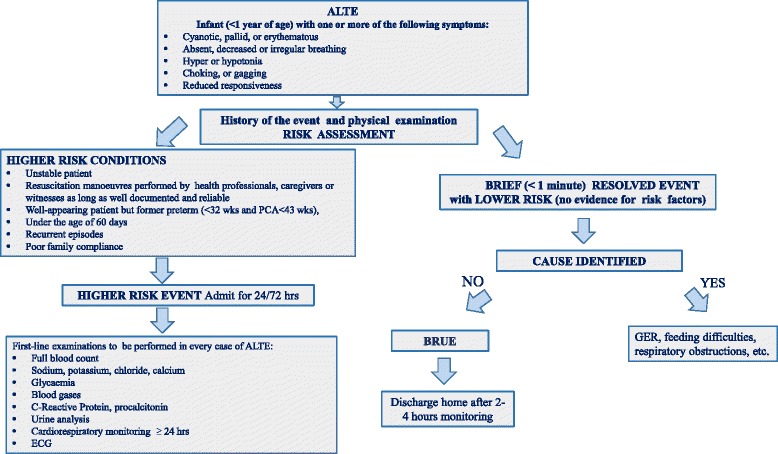
ALTE and BRUE definitions and ALTE first-level examinations

We recommend to carry out at least some basic diagnostic tests on all infants hospitalised for ALTE (Fig. 1).

**Evidence level V**

**Grade of recommendation B**

## Query 7

### What second-level examinations should be performed?

The selection of the second-level examinations should be guided by the history, the physical examination and the results of the first-approach investigations of each individual case.

**Fig. 2:**
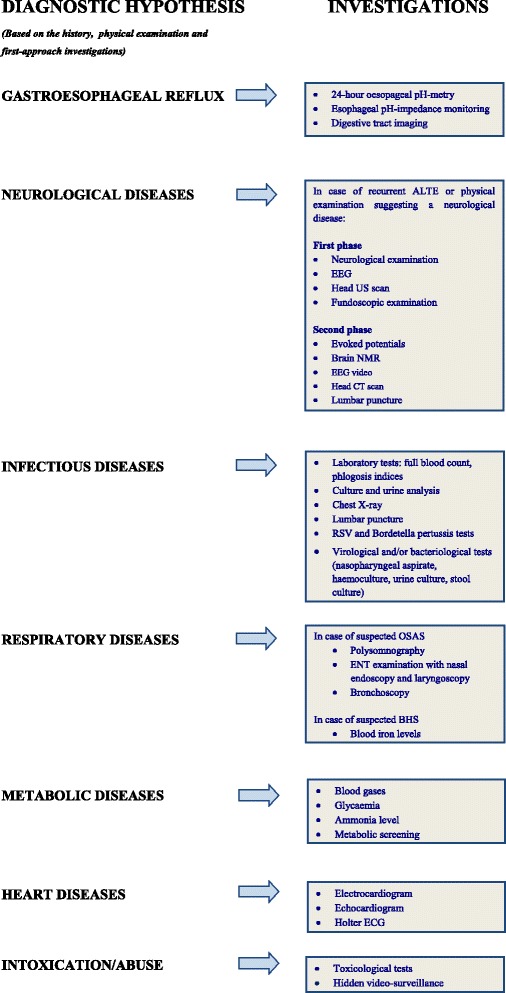
ALTE second-level examinations

The most frequently recommended in literature [14–16, 37, 38, 45, 94] are listed in Fig. 2.

### Recommendation 17

When planning the second-level examinations, a multidisciplinary evaluation is recommended.

**Evidence level V**

**Grade of recommendation B**

## Query 8

### When should an infant with a clinical history of ALTE be hospitalised?

The decision to admit a patient with a history of ALTE to hospital is difficult [17, 95] due to the age of the infant, the anxiety of the family and the unclear history of the episode, which run the risk of either overemphasising or minimising the episode.

Hospitalisation for ALTE is recommended after resuscitation manoeuvres by those providing first aid, especially if healthcare operators, or if the conditions of the infant appear unstable [14, 92], and according to Claudius [95] possibly also in case of prematurity, the age of the infant and the number of episodes of ALTE. In particular, preterm newborns, below 43 weeks PCA, are exposed to the greatest risks of “extreme events” (bradycardia less than 60 bpm for at least 10 s in infants below 44 weeks PCA and below 50 bpm for at least 10 s if at least 44 weeks PCA or apnoea lasting longer than 30 s) [96], and therefore they require more surveillance.

In their study, Mittal and colleagues identified infants with ALTE who are at a higher risk of adverse outcomes and they recorded significant interventions occurring during hospitalization [20].

They mainly considered as “significant interventions”, admission to an Intensive Care Unit (ICU), supplemental oxygen for hypoxia, recurrent episodes of ALTE, discharge with a home apnoea monitor, and concluded that cases of prematurity, abnormal physical examination, colour change to cyanosis, absence of Upper Respiratory Tract Infections (URI) symptoms in the previous 24 h, and absence of choking during the episode, could represent indications for admitting the infant to hospital [20].

Besides the above mentioned risk factors, family compliance should also be specifically considered in order to decide to discharge the infant from the ED [91].

Hospitalization is adviced when the following risk factors are present, even though the scientific evidence is inconsistent.

1. Age < 1 month
2. Preterm birth (PCA < 43 weeks)
3. Family history of Sudden Unexpected Deaths in Infancy (SUDI) or SIDS
4. Unstable at the time of the examination
5. Event not connected to feeding
6. Event during sleep
7. Recurrent ALTE
8. The need for resuscitation
9. Poor family compliance

As a general rule, the episodes which can be considered as less important include those temporally correlated to feeding (within 30 min after the feed), those occurring for the first time, those that occur while awake, those characterised by erythrosis rather than cyanosis or pallor, and those which have resolved spontaneously or after mild stimulation. When these cases are diagnosed as BRUE hospitalization is not recommended, while, if risk factors are present, it is recommended to keep the patient under observation for at least 24 h [46, 96–98].

Finally, when discharging an infant after an episode of ALTE, the risk of recurrence should be considered and the family informed accordingly. In fact, 2.5% of cases require readmission within 30 days after the first episode [17]. A very recent study in Japan demonstrates that respiratory infection symptoms at ED admittance may be independent risk factors for recurrent ALTE [99].

### Recommendation 18

Admission is recommended for at least 24 h in the following cases:

- Infants in clinically unstable conditions or who have undergone resuscitation manoeuvres
- Infants in clinically stable conditions who present one or more of the following features: preterm birth (PCA < 43 sett.), less than 30 days of age, a history of recurrent ALTE, poor family compliance, a family history of sudden deaths.

**Evidence level IV**

**Grade of recommendation B**

## Query 9

### Is hospital cardiorespiratory monitoring always indicated in infants with ALTE?

A cardiorespiratory monitoring device should always be applied to all infants admitted with ALTE at least during the first 24 h. Monitoring reassures parents and identifies relapses that may occur in 30-60% cases of severe ALTE [100–102]. Monitoring should also allow for identifying the cardiorespiratory pattern and must include not only transthoracic impedance, but also pulse oximetry, since it has been highlighted how severe hypoxemia may occur in the absence of prolonged central apnoea [103]. Conversely, it has been demonstrated [93, 104–106] that hypoxemia accompanies the majority of significant events depending on obstructive apnoea. A cardiorespiratory monitor should ideally be fitted with a data recorder, that is, with a memory and relative software, for recording the cardiorespiratory traces before, during and after the alarm, to be downloaded and scored. These devices give us the chance to obtain very important information about the infant’s cardiorespiratory pattern despite not allowing us to perform a complete sleep study for which a PSG is required [104, 107].

The most recent literature [61, 108–110] confirms what has just been outlined above.

### Recommendation 19

In case of hospitalisation it is recommended to carry out the cardiorespiratory and pulse oxymetry monitoring, and if this is not available, then at least pulse oxymetry alone. It is recommended to carry out monitoring for at least 24 h.

**Evidence level III**

**Grade of recommendation A**

## Query 10

### Which are the indications for home monitoring of infants with ALTE?

In early ‘70s, home cardiorespiratory monitoring become very popular in the USA in order to reduce the risk of SIDS by subjecting infants to electronic surveillance during sleep.

Although various studies have analysed the compliance and quality of life of the family of the infant monitored, the duration of the monitoring, and the type of cardiorespiratory events recorded [92, 99, 104, 110–114], no studies have demonstrated their real effectiveness as life-saving devices. Consequently, while home monitoring should not be generically prescribed to prevent SIDS [14, 92, 107] it can be still considered an extension of the diagnostic process.

The only longitudinal case-control study available highlights that the risk of cardiorespiratory events must only be considered significantly higher in preterm newborns up to 43 weeks PCA [93]. In particular, infants with ALTE enrolled in this study did not show a significantly altered cardiorespiratory pattern compared to controls.

Even the more recent studies agree to recommend home monitoring for infants with severe ALTE [107, 109, 115, 116].

The monitoring must in all cases be considered not only as electronic surveillance regarding episodes that could be life-threatening for the infant, but also as a tool that enables the detecting of central apnoea and/or intermittent hypoxemias that could damage the CNS [117].

In any case, the parents of monitored infants must be informed about the recommendations relating to the reduction of the risk of SIDS, as well as the exact functioning of the monitor and its technological limitations. In addition, they must be trained in the cardiopulmonary resuscitation manoeuvres to be performed when necessary [92].

As already mentioned, the monitors should ideally be cardiorespiratory (based on the thoracic impedance) and oxymetric (for detecting the oxygen saturation), equipped with a data recorder and adjustable alarms depending on the various needs and age of the infant [105]. Devices that are sensitive to the infant’s movements are advised against as they do not allow for detecting or recording potentially harmful cardiorespiratory events (false negatives), besides being susceptible to false alarms (false positives) that could have a negative impact on the caregivers [3]. The duration of home monitoring should not be less than 6 weeks, the period of time within which there is the greatest frequency of recurrences [93, 100]. Parents can suspend after 6 weeks of monitoring if no pathological events have been recorded and/or there is documented resolution of the causes that have given rise to the event [14, 115]. Preterm infants with episodes of ALTE should be monitored until the 43rd week of PCA; in case of persistence of the symptoms, it will be necessary to prolong the monitoring for at least another 6 weeks, until their complete remission [92, 93, 115].

### Recommendation 20

Home monitoring prescription is recommended for infants with severe or recurrent ALTE and for symptomatic preterm infants with a clinical history of ALTE, especially with a PCA < 43 weeks.

**Evidence level III**

**Grade of recommendation A**

### Recommendation 21

The duration of home monitoring should be at least 6 weeks, but in the case of preterm infants with ALTE, it must be prolonged for at least until the 43rd week of PCA.

**Evidence level III**

**Grade of recommendation A**

## Query 11

### What is the prognosis for ALTE?

Literature exploring the outcome of ALTE is limited. Nevertheless, the outcome depends on the causes and extent of the event [115]. In fact, infants with ALTE, who suffer from seizures or neurological disorders, those who have required resuscitation manoeuvres, or those who have suffered recurrent episodes, have a higher death rate and a more severe outcome [118]. In Bonkowsky’s retrospective study [43], out of 471 infants with a clinical history of ALTE, 5% had a neurodevelopmental delay or epilepsy. The question remains open as to whether the underlying neurological disease started off as an ALTE, or whether it was the consequence of the episode [119]. Another relevant aspect is represented by the neurological complications of the “shaken baby syndrome”. Besides being an expression of child abuse, retinal bleeding and cerebral injuries [43] may also be the consequence of inadequate attempts at resuscitation [120]. Consequently, correct resuscitation practices should be transmitted to the parents of infants with a higher risk of ALTE or with recurrent episodes.

A recent 5 years follow-up shows that a single episode of ALTE is not predictive of chronic systemic or neurological diseases [121]. It was also emphasised that while the real risk of a recurrence of ALTE cannot be predicted, it is possible, however, to identify subjects at the highest risk of recurrent events, in particular, those subjected to abuse [31, 122, 123].

The overall death rate for ALTE (irrespective of the individual causes) is low (0.2-1.1%) and not connected to risk factors for SIDS [124, 125].

Abuse is a possible cause of death in subjects with a history of ALTE and therefore this must always be taken into close consideration [122].

### Recommendation 22

The prognosis depends on the cause and extent of the event. The most frequently described complications, despite having a relatively low impact, are neurological.

Parents of infants with ALTE should be trained how to perform resuscitation manoeuvres in case of recurrence.

**Evidence level V**

**Grade of recommendation B**

## Query 12

### Is follow-up necessary after an ALTE episode?

Only few studies on long-term follow-up of ALTE are available [126].

Follow-up of infants with ALTE may reveal possible neurological sequelae, detect cases of unrecognised abuse, evaluate the clinical trend over time [15, 31, 43, 47, 108, 126–128] and provide parents with the necessary resuscitation training and re-training, counseling and psychological support [12, 91, 129].

Referral of some cases of ALTE to specialized centres providing a multidisciplinary approach is indicated [130].

### Recommendation 23

A follow-up is always mandatory for the patient with ALTE, who must be looked after in multidisciplinary settings with specific expertise.

**Level of evidence: V**

**Grade of recommendation B**

## Query 13

### Is there a connection between ALTE and SIDS?

Data deriving from a broad retrospective European study indicate that ALTE precedes 10% of the cases of SIDS [131].

Nevertheless, the results of two prospective studies [11, 12] and three retrospective studies [10, 132, 133] lead to the conclusion that SIDS and ALTE are instead two separate phenomena and that ALTE cannot be understood as a precursor of SIDS. The arguments in favour of a distinction between the two phenomena can be summed up as follows:

- the sharp drop in the incidence of SIDS that occurred after the risk reduction campaign which was not matched by an equal drop in the incidence of ALTE;
- the temporal distribution of the two phenomena which is not exactly superimposable, as ALTE occurs before SIDS;
- only a small percentage of ALTE seem to turn into SIDS (<1%);
- the risk factors for ALTE and SIDS are not the same, with the exception of smoking, male gender, very low birth weight and low gestational age.

On the other hand, there is evidence of altered respiratory control during sleep at least in a subset of SIDS cases, in which serotonergic and adrenergic neuronal systems are involved.

Monoamine oxidase A (MAOA) is the enzyme that down-regulates both these neurotransmitters: particular polymorphisms of the gene that codifies for this enzyme could be at the basis of SIDS.

One systematic study on a polymorphism of the MAOA gene was carried out on a large group of patients (156 cases of SIDS and 260 control subjects). The results indicate a relationship between SIDS and a specific MAOA genotype in males, notoriously at a higher risk of SIDS, which seems to influence the serotonergic and adrenergic systems of the brainstem. This locus is the first X-chromosome locus associated with SIDS. As a result, some anomalies of the brainstem probably play a role in a subset of SIDS cases [134].

Therefore, strong doubts surround the relationship between SIDS and ALTE. Nevertheless, the genetic analyses of the L/L 5HTT, MAOA and the dopamine transporter in patients with ALTE of known origin and IALTE, demonstrate a possible association between IALTE and SIDS, in contrast with what has been observed between cases of ALTE of known origin and SIDS.

In short, the two situations (IALTE and SIDS) could represent a different phenotypic expression with a common genetic base [90].

Further studies are necessary before assuming that the two phenomena are merely a different phenotypic expression of the same genetic defect.

### Recommendation 24

A correlation between ALTE and SIDS may be ruled out, therefore, when informing the family about the risk of SIDS after an ALTE, we can be reassuring.

**Evidence level V**

**Grade of recommendation D**

## Query 14

### What does sudden unexpected postnatal collapse (SUPC) mean?

According to the 2011 definition of the British Association of Perinatal Medicine (BAPM), neonatal collapse or SUPC means any newborn in the first week of life with GA > 35 weeks, with a normal Apgar at the 5th minute of life, considered suitable for being normally managed, but which has presented a sudden unexpected cardiocirculatory and respiratory collapse requiring resuscitation with ventilation and leading to death, neonatal intensive care or encephalopathy [135].

This definition also implies that cases of SUPC can be identified as ALTE, when the newborn survives, and as cases of Sudden Unexpected Early Neonatal Death (SUEND) when they lead to death.

The term SUPC refers to a clinical situation that is always severe, therefore excluding mild episodes of cyanosis and apnoea which are simply resolved with aspiration of the airways and tactile stimulation, and that are at times misleadingly cited as ALTE in some works [136].

According to Herlenius [137] 36% of the SUPCs occur during the first 2 h of the newborn’s life, 29% between 2 and 24 h, 24% between 25 and 72 h, and 9% between 4 and 7 days of life. In the case studies collected by Becher [138], the onset of SUPC takes place between 6 and 643 min after birth, on average, at 70 min.

In literature, the incidence range is relatively wide: from 40/100,000 in a Scottish maternity hospital [139] to 2.6/100,000 in Germany [140] and 0.5/100,000 in Australia [141].

This variability may be due to a combination of different degrees of accuracy in the reporting and to different definition.

In 58% of cases [142] a precise cause can be documented: metabolic diseases, congenital malformations, neonatal pulmonary hypertension, and bacterial infections.

The lack of documentation regarding a specific aetiology is very frequent: in 42% of Weber’s cases, in 53% of Pejovic’s cases [143], and up to 67% of Becher’s cases [138].

A post-asphyxia syndrome develops in 73% of cases (22/30) and the outcome is poor in 33% (10/30) of cases, with death (5/30; equal to 16%) or neurological sequelae at 1 year of life [138].

### What are the risk factors for SUPC?

In the subgroup of SUPC without an evident cause (“idiopathic” SUPC) a series of risk factors, are identified, some of which cannot be modified, such as primiparity (OR: 6.22), while others are amenable to modification: the potentially asphyxiating position of the newborn (OR: 6.45) [144], mother in lithotomy position, skin to skin contact, bed-sharing, unsupervised feeding during the first 2 h of life and lastly maternal distraction (possibly also due to the use of a mobile phone for talking or sending text messages) [143].

Becher stresses that 80% of idiopathic SUPC are associated with an obstruction of the airways due to inappropriate prone position and/or the type of feeding.

### How the risk of SUPC can be reduced?

Unfortunately, current literature does not indicate any effective interventions to prevent SUPC. Moreover, neither position statements on the topic nor clear recommendations by the scientific societies are available, with the exception of the BAPM document [135].

Even the recent document of the Task Force on SIDS of the American Academy of Pediatrics does not add much to this issue [145], simply warning that inappropriate skin-to-skin contact and rooming-in can be associated with SUPC. Rooming-in may be of concern particularly when mother and baby are sleeping together in the mother’s bed.

The benefits of skin-to-skin contact in the delivery room are well known: “humanization” of care for the mother-infant pair, promotion of the mother-baby relationship, promotion of breastfeeding, colonization of the newborn with maternal bacteria, influence on the composition of the intestinal microbiota of the newborn. However, in recent years there has been great emphasis on the need for skin-to-skin contact to be carried out safely, avoiding asphyxiating positions of the newborn, improving supervision by the healthcare personnel, increasing the attention paid by the family to the baby.

The safety of rooming-in and bed-sharing in the context of the prevention of SUPC is debatable. Difficulties faced in implementing and maintaining good standards are well known. Moreover, the risk of the infant falling out of bed while bed-sharing still represents a nightmare for postnatal health workers as well as for families.

In other words, there is a persistent need to integrate safe skin-to-skin contact and rooming-in (representing respectively Step 2 and Step 7 of the *Baby Friendly Hospital Initiative)*, with safe postnatal care.

The Guidelines for Perinatal Care of the AAP-ACOG [146] recommend a physical examination and observation of the newborn during the first 6-12 h of life, i.e. during the stabilization-transition period.

Currently, in order to reduce the risk of SUPC during the first 2 h of life (when the cases of SUPC are concentrated), different strategies have been implemented that share the following interventions: 1) non-intrusive *supervision* of the mother-infant pair by health professionals, avoiding positions at risk of asphyxia, and 2) *information* for parents regarding the signs of the infant’s wellbeing, aimed at involving parents in controlling the newborn infant in the early postnatal period [137].

In this regard, education programs for families are advised.

More and more maternity hospitals worldwide have drawn up checklists as a tool for assessing the newborn during the first 2 h after birth, when the risk of SUPC is higher.

More specifically, two checklists have been published in literature: 1) the first by Ludington-Hoe & Morgan in Cleveland [147] and the second by Davanzo at the Istituto Materno-Infantile IRCCS Burlo Garofolo, in Trieste [148] (Table 5).

**Table 5:**
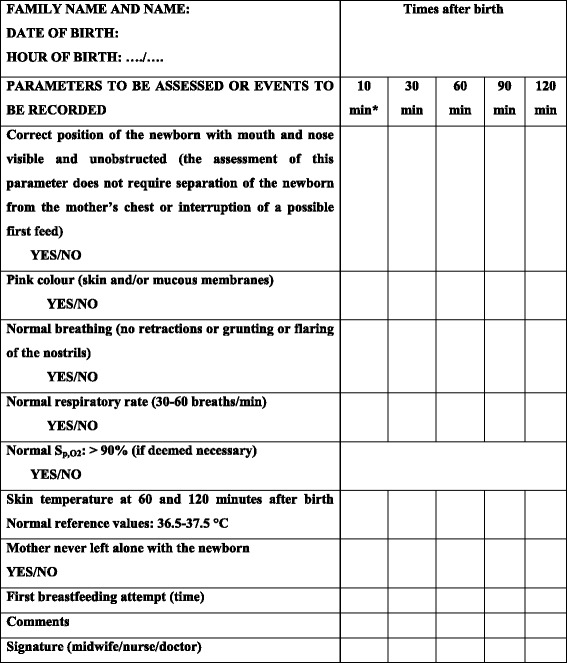
CheckCe, particularly during the skin to skin contact [148]

Position: Prone position of the newborn during skin-to-skin contact (SSC) should not obstruct the airways or prevent adequate breathing. The healthcare professional should intervene to correct at risk positions

Colour: Areas to be examined are lips and tongue. Assess the presence of abnormal skin colour: cyanotic or pale

Breath: Breathing pattern, respiratory rate, and amplitude of breaths are physiologically irregular in the newborn. The presence of the following abnormal conditions must be assessed: (a) mouth or nose obstruction; (b) respiratory rate > 60 breaths/min or <30 breaths/min; (c) apnoea or dyspnoea (flaring of the nostrils, grunting, or intercostal retractions)

Pulse oximetry: This should be measured only if the infant shows some abnormal signs during surveillance, appears “unconvincing” to the observer, or if judged appropriate in the local settings due to staff shortage. For term newborns, a value higher than 90% (10° percentile) is considered normal after the first 10 min of life

Skin temperature: Normal skin temperature is 36.5 °C-37.5 °C

Mother and infant should never be left alone: mother-infant SSC should be supervised periodically (at 30, 60, 90, and 120 min of life) by midwives. Presence of the father or another person should be guaranteed. The room should be adequately lit, so that the baby’s skin colour can be seen. Should the mother appear fatigued or drowsy, consider placing the infant in his or her own crib or have the father hold him or her. Emergency call procedures should be clearly explained to parents. If no surveillance can be provided and the parents’ reliability cannot be assessed, the safest option is to admit the newborn infant to the regular nursery. It is recommended to turn off mobile phones to avoid a well-known and relevant source of distraction

These checklists record some items relevant to the protection and safety of the newborn: the correct position on the mother’s chest, the presence of at least a second person in addition to the mother in the delivery room, the monitoring of neonatal parameters such as skin colour, respiratory rate, presence of dyspnoea, temperature.

A local decision can be made also to include in these surveillance grids the routine use of pulse oximetry for detecting the S_p,O2_ and the heart rate. Nevertheless, we must recognize that at the moment, no clear cost/benefit evaluation is available regarding the implementation of pulse oximetry in preventing SUPC. Certainly, when routinely used, pulse oximetry could represent a medicalising intervention. Although it could interfere with the mother-baby relationship and the natural childbirth, it can be considered in case of staff shortage.

Pending documentation on the effectiveness of these checklists to prevent SUPC in the early postpartum period, the application of these checklists in the delivery room should be considered a best practice.

Checks of the newborn’s conditions are foreseen and conducted by the midwife or the nurse, or paediatrician/neonatologist at 10, 30, 60, 90, and 120 min after birth. The checks are based on the assessment of certain selected vital parameters for ascertaining whether the safety conditions are present (YES) or absent (NO). The healthcare operator identifies him/herself by signing at the bottom.

During the remaining part of hospitalization after childbirth, the information to parents is still crucial, including the advice that healthy newborn infants should be put back to sleep in her/his cot in the rooming-in settings. The use of a patient safety contract, particularly for the high risk situations (e.g maternal obesity and tiredness), has been suggested by the Task Force on SIDS of the AAP [145].

Certainly, surveillance by a nurse/midwife is needed, particularly during the transition period (first 6-12 h) and with special focus on the healthy late-preterm infants and ultimately until the newborn infant demonstrates to be stable [149]. However, we must recognize that minimum requirements for staffing in order to improve safety have not yet been clearly defined at an international level.

Currently, the indication for monitoring every 30 min during night-time and early morning for high risk, even healthy, dyads [145] can be sustained in some American hospitals that can afford to provide a dyads/nurse ratio of no more than 3:1 [150], but it is far from being achieved in maternity hospitals in Italy, and possibly also other European countries. Moreover, it is not only an issue of affordability, but also the possible intrusiveness of the medical care in a context of unproven impact of a stricter versus a looser surveillance pattern.

### Workup after SUPC

Newborns with postnatal collapse should receive intensive care and subsequently be fully investigated for possible common causes, specifically:

1. congenital infections;
2. congenital abnormalities (particularly cardiac anomalies);
3. respiratory conditions;
4. anaemia;
5. hypoglycaemia;
6. congenital adrenal hyperplasia;
7. neurological and neuromuscular disorders;
8. metabolic diseases.

A valid clinical orientation has been provided by the BAPM guidelines [135], that propose a protocol aimed at collecting information as well as performing investigations on the event.

The staff should complete a full parental medical history, including the use of alcohol and drugs, smoking, obstetric history (including infections, foetal growth and anomalies), the type of feeding, and the circumstances surrounding the SUPC (witnesses, position of baby, etc.).

In addition to an exhaustive list of possible tests, the paediatrician should decide on an individual basis, which tests should be carried out to appropriately investigate the case with the least amount of intervention (Table 6). Consequently, Table 6 cannot be considered the routine workup, but simply as a choice list.

**Table 6:** Possible investigations following a case of SUPC [135]

1.	Placenta pathology and microbiology		
2.	Maternal blood: Kleihauer (in case of anaemia), viral titres (if not previously performed)		
3.	Maternal vaginal swabs (if not previously performed)		
4.	Neonatal blood:		
	4.1	Full blood count, coagulation, blood gas, renal and liver biochemistry, glucose, lactate, calcium, magnesium, ammonia, beta-hydroxybutyrate, amino acids, free fatty acids, acylcarnitine profile, urate, uric acid, culture, viral titres, blood spot for cardiolipin analysis	
	4.2	Genetic testing:	
		4.2.1	In case of suspicion of unrecognised hypoventilation /apnoea, a sample of DNA should be taken specifically to search for abnormalities of the PHOX2B gene
		4.2.2	Testing for mutations and copy number variation in MECP2 should be considered as it may be revealed as newborn encephalopathy and/or apnoeas and respiratory collapse
		4.2.3	Array-based comparative genomic hybridisation is a useful investigation that will replace conventional karyotyping in the near future as a method for detecting causative chromosomal deletions and duplications
5.	Cerebrospinal fluid: biochemistry, culture, virology, lactate, amino acids including glycine		
6.	Urine: virology, toxicology, organic acids including orotic acid, amino acids including urinary sulphocysteine and urine to be retained for storage		
7.	Imaging: cranial ultrasound scan, MRI, renal/adrenal ultrasound scan, echocardiogram		
8.	ECG		
9.	Ophthalmoscopy		
10.	Skin biopsy for fibroblast culture		
11.	Muscle biopsy if unable to exclude neuromuscular or mitochondrial disorder		
12.	EEG		

In the case of fatal SUPC or SUEND, an autopsy is strongly recommended, according to the Italian decree “Diagnostic protocols in cases of sudden infantile death and unexpected death of the foetus” (14A08847) (GU General Series no. 272 of 22-11-2014 – Ordinary Supplement no. 89).

### Aiming at preventing SUPC: A summary

In order to prevent neonatal collapse, greater attention should be paid in the case of primiparity, operative vaginal delivery, maternal sedation, maternal fatigue and maternal distraction. The usual position of the newborn in the delivery room during maternal skin-to-skin contact is the prone one, which represents an exception to the general rule that recommends the supine position for the newborn’s safety. The supervision by health professionals, together with that of the parents during skin-to-skin contact in the delivery room should therefore be very strict, in particular avoiding and/or correcting potentially asphyxiating positions.

After the first hours of life, parents should be instructed how to avoid the prone position or a potentially asphyxiating position, that precludes the patency of the mouth and nostrils. The issue of keeping the newborn’s safety during the post-partum period must be part of the routine written and verbal information transmitted to parents during pregnancy as well as during the postpartum period. Monitoring the conditions of the newborn must be implemented, especially during the first 2 h of life.

Simple checklists as a tool for assessing the newborn during the first 2 h after birth are available.

During the remaining part of hospitalization after childbirth, non-intrusive surveillance is needed. The information to parents is still crucial, including the recommendation that while rooming-in, newborns should be put back to sleep in their cot. Midwives/nurses should strictly observe healthy newborn infants during the transition period (first 6-12 h after birth). Particularly, between 3 and 12 h after birth, at least 2 observations should be made. After the transition period or after the first 12 h of life, mother-baby dyads should be observed at least once every 8 h until hospital discharge. Bed-sharing should be discouraged. Whenever parents give preference to bed-sharing, they should be advised that it is generally contraindicated and particularly in case of obesity, sedation and tiredness.

### Recommendation 25

In order to prevent neonatal collapse, skin to skin contact in the delivery room should be implemented by health professionals, using available checklists for supervision.

Parents and accompanying persons should be informed and involved on the appropriate way to keep skin to skin contact between mother and baby as safe as possible for the newborn infant in the delivery room as well as subsequently in the postpartum ward.

Health professionals should discourage bed sharing and strictly observe healthy newborn infants during the transition period and regularly until hospital discharge.

**Evidence level V**

**Grade of recommendations B**

## Conclusions

The aim of these GL is to provide an updated, multidisciplinary document on the clinical management of ALTE. This topic is of special interest because in case of ALTE the physician is called on to promptly decide which exams need to be carried out, whether the infant should be kept under observation, and whether the family will be capable of managing the infant at home, even in the case of an “apparently” mild event.

Recently, the AAP published the GL on BRUE [6] in which they only refer to mild idiopathic cases, having decided to abolish the term ALTE. In our document we have maintained the acronym ALTE, reserving it for serious cases that are still unexplainable after first and second level examinations. In the events for which the aetiology is ascertained we use this acronym to describe the common symptoms at the onset, however, depending on the case, the final diagnosis may be seizures, gastroesophageal reflux, infection, arrhythmia, etc. Lastly, we address the emerging problem of so-called neonatal ALTE, also known as SUPC, and provide the recommendations for safe management of skin-to-skin contact. We trust that these GL will be clinically helpful and make it possible to safely manage infants affected by ALTE, while also reducing family stress and ensuring correct use of the available resources.

## Abbreviations

5HTT: _Serotonin transporter_
AAP: American Academy of Pediatrics
AAPACOG: American Academy of Pediatrics, American College of Obstetricians and Gynecologists
ALTE: Apparent Life-Threatening Event
ANS: Autonomic Nervous System
BAPM: British Association of Perinatal Medicine
BHS: Breath Holding Spells
BRUE: Brief Resolved Unexplained Events
CCHS: Congenital Central Hypoventilation Syndrome
CNS: Central Nervous System
CRP: C-reactive protein
DPPIV: Dipeptidyl peptidase IV
ECG: Electrocardiogram
ED: Emergency Department
EEG: Electroencephalogram
ENT: Ear, Nose and Throat
ESPGHAN: European Society for Paediatric Gastroenterology Hepatology and Nutrition
GER: Gastroesophageal reflux
GERD: GER-disease
GL: Guidelines
IALTE: Idiopathic ALTE
ICU: Intensive Care Unit
MAOA: Monoamine oxidase A
MRI: Magnetic Resonance Imaging
NICE: The National Istitute for Health and Care Excellence
OSAS: Obstructive Sleep Apnoea Syndrome
PCA: Post-conceptional age
PNLG: National Guidelines Plan
PSG: Polysomnography
P_tc,O2_: Transcutaneous Oxygen Tension
RSV: Respiratory Syncytial Virus
SIDS: Sudden Infant Death Syndrome
SIP: Italian Paediatrics Society
S_p,O2_: Oxygen Saturation
SRE: Spontaneous Respiratory Events
SUDI: Sudden Unexpected Deaths in Infancy
SUEND: Sudden Unexpected Early Neonatal Death
SUPC: Sudden Unexpected Postnatal Collapse
URI: Upper Respiratory Tract Infections
UTIs: Urinary Tract Infections
Vmax FRC: Maximum flow at functional residual capacity
WBC: Whole Blood Count
